# Exome Enrichment and SOLiD Sequencing of Formalin Fixed Paraffin Embedded (FFPE) Prostate Cancer Tissue

**DOI:** 10.3390/ijms13078933

**Published:** 2012-07-17

**Authors:** Roopika Menon, Mario Deng, Diana Boehm, Martin Braun, Falko Fend, Detlef Boehm, Saskia Biskup, Sven Perner

**Affiliations:** 1 Department of Prostate Cancer Research, Institute of Pathology, University Hospital of Bonn, Bonn 53127, Germany; E-Mails: mroopika@gmail.com (R.M.); mariodeng@googlemail.com (M.D.); dianaboehm@web.de (D.B.); martin.braun85@googlemail.com (M.B.); 2 Department of Hematology and Oncology, University Hospital of Tuebingen, Tuebingen 72076, Germany; E-Mail: falko.fend@med.uni-tuebingen.de; 3 Center for Genomics and Transcriptomics, CeGaT GmbH, Paul-Ehrlich-Str.17, Tuebingen 72076, Germany; E-Mails: detlef.boehm@cegat.de (D.B.); saskia.biskup@cegat.de (S.B.)

**Keywords:** exome sequencing, SOLiD4, prostate cancer, next-generation sequencing

## Abstract

Next generation sequencing (NGS) technologies have revolutionized cancer research allowing the comprehensive study of cancer using high throughput deep sequencing methodologies. These methods detect genomic alterations, nucleotide substitutions, insertions, deletions and copy number alterations. SOLiD (Sequencing by Oligonucleotide Ligation and Detection, Life Technologies) is a promising technology generating billions of 50 bp sequencing reads. This robust technique, successfully applied in gene identification, might be helpful in detecting novel genes associated with cancer initiation and progression using formalin fixed paraffin embedded (FFPE) tissue. This study’s aim was to compare the validity of whole exome sequencing of fresh-frozen *vs*. FFPE tumor tissue by normalization to normal prostatic FFPE tissue, obtained from the same patient. One primary fresh-frozen sample, corresponding FFPE prostate cancer sample and matched adjacent normal prostatic tissue was subjected to exome sequencing. The sequenced reads were mapped and compared. Our study was the first to show comparable exome sequencing results between FFPE and corresponding fresh-frozen cancer tissues using SOLiD sequencing. A prior study has been conducted comparing the validity of sequencing of FFPE *vs*. fresh frozen samples using other NGS platforms. Our validation further proves that FFPE material is a reliable source of material for whole exome sequencing.

## 1. Introduction

The recent development in next generation sequencing (NGS) technologies has revolutionized cancer research [1–4]. Next generation sequencing (NGS) technologies has revolutionized cancer research by making it possible to comprehensively study the complexity of cancer using high throughput deep sequencing methodologies. These methods enable the detection of genomic alterations, nucleotide substitutions, insertions, deletions and copy number alterations [5,6]. Recently, whole genome sequencing was performed on a set of seven fresh frozen prostate cancer (PCa) samples to study the genomic complexities involved in localized PCa [7]. Similarly, another study described the mutation frequencies observed in advanced and lethal prostate cancer by exome sequencing of xenograft tissue [8]. Studies in this field are limited due to the high cost of NGS, and the challenge involved in data analysis, requiring time and bioinformatic expertise [9]. Another major limiting factor of this technology is the scarcity of fresh frozen specimen having a high-grade DNA integrity. On the other hand, the use of formalin fixed paraffin embedded material (FFPE) from pathology archives would open up the treasure of abundantly available patient material for sequencing. Despite the known adverse effect of formalin fixatives on the content and integrity of nucleic acids [10–12], previous studies have successfully used FFPE tissue samples for copy number analysis, mutation analysis, and for the determination of germline variations using the Illumina platform [13,14].

To assess the integrity of the FFPE tissues, we performed whole exome sequencing of fresh-frozen and FFPE tumor tissue by normalization to normal prostatic FFPE tissue, all obtained from the same patient. We used the SOLiD4 (Sequencing by Oligonucleotide Ligation and Detection, Life Technologies) sequencing platform, known to have an accuracy of 99.94% to detect single nucleotide variations (SNVs). The advantage of using the SOLiD4 platform is the generation of billions of 50 bp sequencing reads. These are optimal for sequencing the FFPE tissue that is degraded to a certain extent. We further evaluated the reproducibility of the sequencing data using two different fixation methods (fresh frozen and FFPE) of the same sample, in order to see if FFPE tissue could be used as a promising alternative to fresh frozen samples for SOLiD NGS technologies.

## 2. Results and Discussion

### 2.1. Results

#### 2.1.1. Exome Sequencing of FFPE and Fresh Frozen Prostate Cancer Tissue

The sequencing output resulted in approximately 99 to 113 million reads for each of the three samples, with a sequencing coverage of 50x. Out of these, the uniquely mapped reads for each sample were in the range of 43.9 million for FFPE normal prostatic, 42.5 million for FFPE tumor and 51.0 million for fresh frozen tumor. The percentage of on-target reads for each sample was 81.9% for FFPE normal prostatic, 78.47% for FFPE tumor and 82.44% for fresh frozen tumor. With reference to all the on-target reads, the percentage of targeted exons for each sample was 98.19% for FFPE normal prostatic, 98.32% for FFPE tumor and 98.52% for fresh frozen tumor (Table 1). The average coverage for the SNV analysis was ~86x.

The high mapping stringency approach generated reliable values for the unique placed reads. Our results show similar on-target reads within ± 150 bp for each sample, including 81.91% for the FFPE normal prostatic, 78.47% for the FFPE tumor and 82.44% for the fresh-frozen tumor. We detectably captured 98.19% of targeted exons for the FFPE normal prostatic, compared to the 98.32% for the FFPE tumor and 98.52% for the fresh-frozen tumor tissue.

#### 2.1.2. SNV Analysis

**Figure 1:**
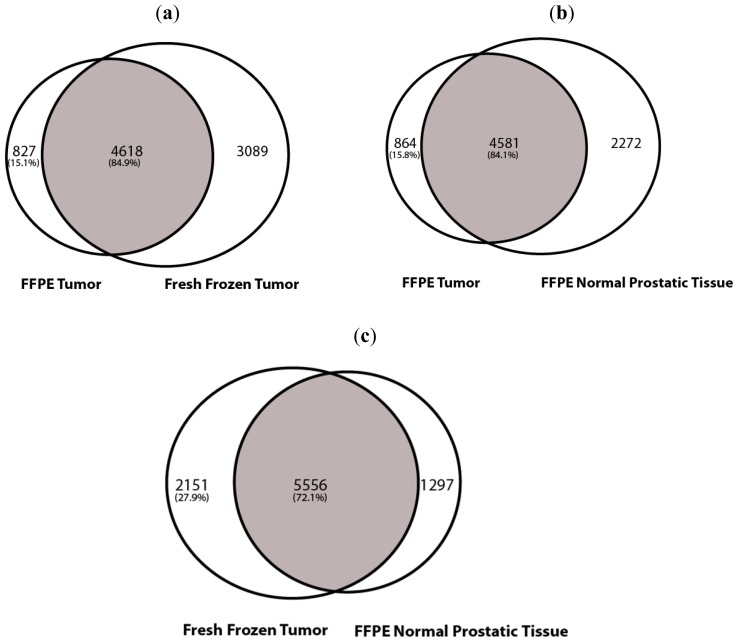
Venn diagrams representing single nucleotide variation (SNV) profiles. The SNV analysis showed a total of 6853 SNVs for FFPE normal prostatic, 5445 SNVs for FFPE tumor and 7707 SNVs for fresh frozen tumor. (**a**) FFPE tumor *vs*. fresh frozen tumor tissue: The total number of common SNVs between FFPE tumor and fresh frozen tumor were 4618. 84.9% of the FFPE SNVs were common to the fresh frozen sample. (**b**) FFPE tumor *vs*. FFPE normal prostatic tissue: Upon normalization with FFPE normal prostatic, the tumor specific SNVs for FFPE tumor were 864. There was a 84.1% overlap between the fresh frozen tumor and FFPE non prostatic tissue. (**c**) Fresh-frozen tumor *vs.* FFPE normal prostatic tissue: Upon normalization with FFPE normal prostatic, the tumor specific SNVs for fresh frozen tumor were 2151. There was a 72.1% overlap between FFPE tumor and FFPE normal prostatic tissue.

The SNV analysis showed a total of 6853 SNVs for FFPE normal prostatic, 5445 SNVs for FFPE tumor and 7707 SNVs for fresh frozen tumor. The total number of common SNVs between FFPE tumor and fresh frozen tumor were 4618. Eighty four point nine percent of the FFPE tumor SNVs were common to the fresh frozen sample. The tumor tissue was normalized with non-tumor tissue from the same patient in order to disregard the common SNPs also seen in the non-tumor tissue. Upon normalization with FFPE normal prostatic, the tumor specific SNVs for FFPE tumor were 864 and for fresh frozen tumor were 2151 (Figure 1). Similarly, there was a 84.1% overlap between the FFPE tumor and FFPE normal prostatic tissue and 72.1% overlap between the fresh frozen tumor and FFPE normal prostatic tissue. Using stringent SNV calling at the regions of interest, we mapped 4227 SNVs common to all three data sets. By comparing the SNV profiles between tumor and normal prostatic sets, 391 SNVs were found to be specific to the tumor tissues. In order to detect the false positive rate in our sequenced sample, we set the SNVs called by the fresh frozen sample as standard and detected the false positive rate by calculating the number of SNVs present in the FFPE tissue but not seen in the fresh frozen tissue. The false positive rate was approximately 10%. Similarly, upon looking at the transversion and transition mutations in the samples, we noticed that the FFPE tumor and the FFPE normal prostatic tissue possess approximately 70% of transition mutations and 30% of transversion mutations. On the other hand, the fresh frozen sample shows 96% of transition mutations and 4% of transversion mutations. The drastic increase in the percentage of transversion mutations could be attributed to the artifacts generated by the formalin fixation, which introduces cross-linking between cytosine nucleotides.

#### 2.1.3. Copy Number Variation (CNV Analysis)

**Figure 2:**
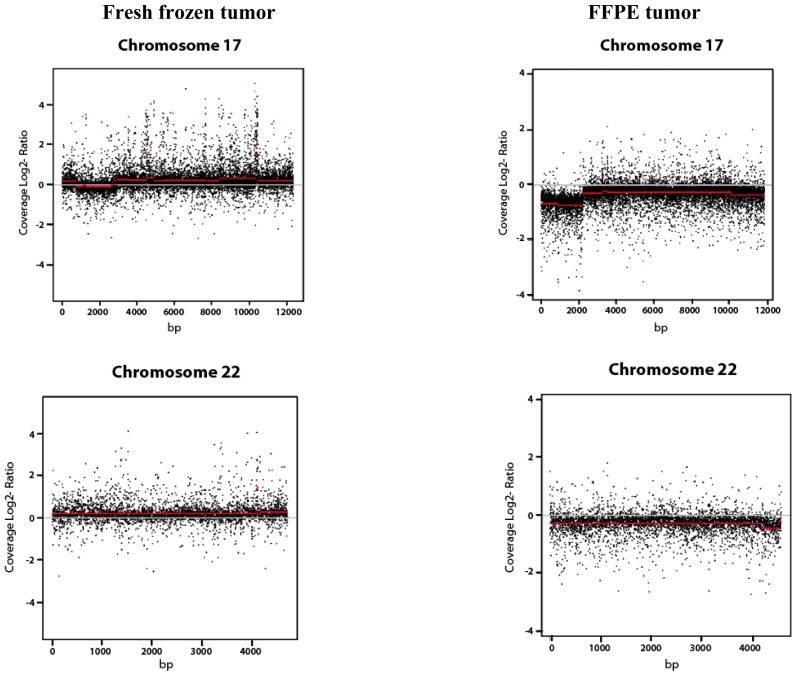

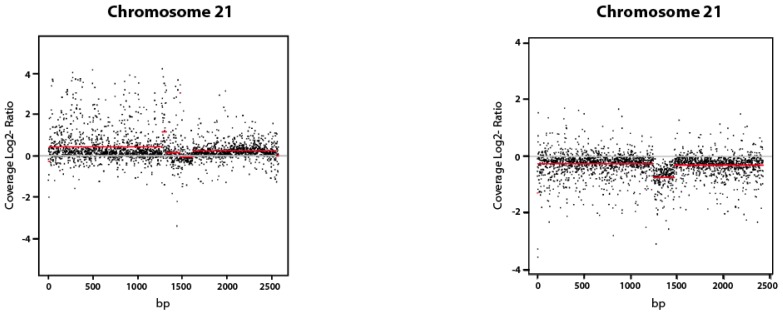
Copy number variation analysis of exome sequenced fresh frozen tumor and FFPE tumor after normalization with FFPE normal prostatic tissue. Copy number variation analysis of fresh frozen tumor and FFPE tumor *versus* FFPE normal prostatic on three chromosomes. For each window the ratio between normal and tumor tissue has been calculated. Furthermore, the log2 has been applied to these ratios. The genomic position is represented on the *x*-axis. The log2 ratios of the fragments are represented on the *y*-axis. The average is depicted by the red line.

We studied the copy number variation between the FFPE fixed and the fresh frozen tumor sample, both obtained from the same patient. Each tumor sample was normalized using normal prostatic FFPE fixed tissue obtained from the same patient. The copy number variation observed after normalization with normal prostatic was then plotted (Figure 2).

### 2.2. Discussion

Next generation sequencing technologies have emerged as a powerful tool to study the genomic and transcriptomic alterations involved in cancer [15]. These techniques have revolutionized cancer research by making it possible to comprehensively study the complexity of cancer using high throughput deep sequencing methodologies. These methods enable the detection of genomic alterations, nucleotide substitutions, insertions, deletions and copy number alterations. Most commonly, fresh frozen tissue is used for NGS due to superior molecular integrity and the absence of fixatives. Previous sequencing studies have shown that fresh frozen material could be used as a quality indicator, as the integrity of the DNA and RNA is high [16]. However, fresh frozen material is rare and complex in terms of storage and handling. FFPE, on the other hand, is commonly archived in the pathology departments for thousands of patients with detailed clinical data with follow-up data available. The material is easily available and also easy to handle, unlike fresh frozen material. Consequently, FFPE tissue could be a reliable source of sequencing material. We have investigated the exome sequencing efficiency of FFPE material compared to fresh frozen material using the SOLiD4 sequencing platform.

To validate the sequencing efficiency of FFPE tissue, we performed an SNV and CNV analysis. Overall, there was a major overlap between the SNVs identified in the FFPE tumor and the fresh frozen tumor tissue (Figure 1). In our data, we also observe a larger number of SNVs for the fresh frozen material when compared to the FFPE material. It is known that fresh frozen material is considered to be state of the art material for sequencing and therefore we expect the tissue to be intact and have a larger number of valid SNV calls. Similarly, due to the formalin fixation, the FFPE samples do tend to have a larger degree of fragmentation. This fragmentation results in non-uniquely aligned reads, which in turn gets automatically removed during the alignment procedure. Therefore, the total SNV calls for the FFPE tissue is relatively less than that of the fresh frozen sample; and when aligning both the tumor tissues with the FFPE normal prostatic sample, this results in a larger number of tumor specific SNVs for the fresh frozen compared to the FFPE tumor tissue.

The CNV analysis showed that the plots between both the FFPE and fresh frozen tumor samples varied to a certain degree. The CNV plots depict an extent of degradation in the fresh frozen tumor sample. Due to the lack of fresh frozen normal prostatic tissue, we used FFPE normal prostatic tissue for normalization purposes. The noise in the CNV plot of the fresh frozen tumor sample may have been caused due to the normalization of the sample with FFPE normal prostatic tissue. The FFPE tissue may have been degraded due to the formalin fixation protocol and the long-term room temperature storage of the FFPE sample. For CNV analysis, an even higher sequencing coverage would be required to achieve better results [16]. Due to the high degree of formalin-induced fragmentation, a high sequencing coverage would generate reads that are specific when mapped to the human genome.

Using FFPE tissue for sequencing will lead to unlocking the treasure of tissues hidden in pathology archives. There are many advantages of using FFPE tissue. While performing sequencing, FFPE normal prostatic material used for normalization purposes would yield the expected results, as the availability of matched normal prostatic fresh frozen material is limited. Using FFPE tissue for sequencing would definitely provide a better understanding of the functional biology of cancer, cancer progression and targeted drug therapy.

A limitation, however, is the high degradation of RNA in FFPE material. Unfortunately, this limits the use of formalin fixed material to only DNA related sequencing protocols, *i.e.*, exome sequencing and targeted sequencing. Transcriptome sequencing still remains a challenge using FFPE material.

## 3. Experimental Section

### 3.1. Tissue Storage

Prostatectomy material was obtained from a patient treated for localized prostate cancer at the University Hospital of Tuebingen, Germany under an Institutional Review Board approved protocol (395/2008BO1). The cancer tissue was cut into two equal parts. Subsequently, one portion was fixed in 10% neutral buffered formalin and embedded in paraffin and the other portion was fixed using the cryo-conservation method. For the same patient, matched normal prostatic tissue was also fixed using the FFPE protocol.

### 3.2. DNA Extraction and Preparation

The FFPE normal prostatic, FFPE tumor and fresh frozen specimen were cut into 3 μm thick sections and stained with hematoxylin and eosin. The sections were assessed by a pathologist (S.P.) to identify the tumor region (tissue containing > 80% tumor cells) or the absence of tumor (*i.e.*, normal prostatic tissue). A 3 mm biopsy needle was then used to punch three cores from each sample for DNA extraction. Core punches, restricted to the tumor region, were performed rather than tissue sections to maintain the homogeneity of the tumor sample. The three cores for each sample were pooled and DNA isolation was performed using the RecoverAll™ Total Nucleic Acid Isolation Kit (Ambion) for the FFPE samples and the PureLink™ Genomic DNA Kit for the fresh frozen specimen.

### 3.3. Library Construction

Three micrograms of each DNA sample was treated to obtain the SOLiD pre-capture library according to the manufacturer’s protocol (Applied Biosystems, Inc.). DNA was sheared using the Covaris S2 to produce fragments with a base pair target range of 150–180 (Covaris, Inc.). The fragments were end repaired and purified using the SOLiD Library Column Purification Kit (Applied Biosystems). The resulting blunt-ended fragments were then ligated to P1 and P2 adaptors. The desired fragment lengths were obtained by running the samples through precast gels (E-Gel SizeSelect 2%, Invitrogen by Life Technologies) for size selection. The purified adapter ligated fragments were subjected to nick translation and library amplification using Platinum PCR Amplification Mix (Invitrogen by Life Technologies) and P1 and P2 amplification primers with 12 amplification cycles, to obtain the genomic pre-captured library.

The libraries were quantified using the Agilent Bioanalyzer DNA 1000 chip and the High-Sensitivity DNA chip.

### 3.4. Targeted Capture and Exome Sequencing

Targeted enrichment was performed with an ABI SOLiD optimized SureSelect human whole exome kit (Agilent SureSelect All Exon G3362, version 1, ELID 027495). The kit is designed to enrich for 165,637 exons (~18,000 genes) covering a total of 37-Mb genomic sequences. Capture libraries were hybridized in solution according to the SureSelect Target Enrichment protocol for the Applied Biosystems SOLiD System (SureSelect Human All Exon Kit and Custom Designs; Version 1.5.1, April 2010). The prepared exome library was further used for emulsion PCRs following the manufacturer’s instructions (Applied Biosystems SOLiD™ 4 System Templated Bead Preparation Guide by life technologies, March 2010) based on a library concentration of 0.5 pM. For each sample, one quad of a SOLiD sequencing slide (Life Technologies) was processed for sequencing as single-end 50-bp reads. Approximately 6 to 8 Gbs of sequence was generated per capture library on the SOLiD4 system.

### 3.5. Bioinformatic Analyses

The sequencing color space reads were mapped to the reference human genome (UCSC hg19) using the BLAT-like fast accurate search tool (BFAST, v0.6.5a) [17]. SAM-files have been filtered using SAMTools (v0.1.15) [18] with the following criteria: PHRED-like consensus of ≥ 30, removal of PCR duplicates. Using the GATKs (v1.1-37-ge63d9d8) [19] default pipeline, a realignment was performed. SNVs were subsequently called using GATK, using default parameters [20]. Also a SNV had to be present in ≥ 25% in the reads at the position, to be declared as an SNV.

### 3.6. Determination of Copy Number Variations

Comparing the FFPE tumor sample against FFPE normal prostatic sample and fresh frozen tumor against FFPE normal prostatic sample, the copy number variation analysis was performed. For this a window of length 350 has been shifted over each position of the samples. For each window the ratio between normal and tumor tissue has been calculated. Furthermore, for normalization purposes, the log2 has been applied to these ratios.

For prioritization and annotation snpEff (v2.0.4) [21] has been used, using default parameters and including ENSEMBLE release 64.

## 4. Conclusions

In conclusion, this is the first study analyzing the reliability and efficiency of using FFPE tissue for exome sequencing with the SOLiD4 sequencing platform. The SNVs and mutations were detected in the FFPE material. The possibility of using FFPE material for next generation sequencing protocols would hasten the process of studying the genetic architecture of various cancers. Furthermore, FFPE sequenced material could also be used in routine diagnostics for the quick and easy detection of prognostic genes.

## Figures and Tables

**Table 1:** Comparison of exome sequence read data between formalin fixed paraffin embedded (FFPE) normal prostatic, FFPE tumor and fresh-frozen tumor tissues.

Parameters	FFPE non-tumor	FFPE tumor	Fresh-frozen tumor
**Reads**	**100,675,212**	**99,910,447**	**113,195,686**
Rejected reads	9,011,246	10,280,465	7,809,343
Valid reads	91,663,966	89,629,982	105,386,343
Non-uniquely mapped reads	47,691,531	47,113,995	54,345,991
Uniquely mapped reads	43,972,435	42,515,987	51,040,352
On-target reads (within ± 150 bp)	36,016,954	33,363,890	42,077,913
On-target reads in%	81.91%	78.47%	82.44%
**All Intervals/Exons**	**165,481**	**165,481**	**165,481**
Targeted exons	162,490	162,699	163,027
Targeted exons in%	98.19%	98.32%	98.52%
Non-targeted exons	2,991	2,782	2,454
Total number of non synonymous SNVs	6,853	5,445	7,707
